# Role of High Mobility Group Box 1 (HMGB1) in SCA17 Pathogenesis

**DOI:** 10.1371/journal.pone.0115809

**Published:** 2014-12-30

**Authors:** Li-Ching Lee, Chiung-Mei Chen, Pin-Rong Wang, Ming-Tsan Su, Guey-Jen Lee-Chen, Chun-Yen Chang

**Affiliations:** 1 Department of Life Science, National Taiwan Normal University, Taipei, Taiwan; 2 Department of Neurology, Chang Gung Memorial Hospital, Chang-Gung University College of Medicine, Taipei, Taiwan; 3 Science Education Center, National Taiwan Normal University, Taipei, Taiwan; Cincinnati Children's Hospital Medical Center, United States of America

## Abstract

Spinocerebellar ataxia type 17 (SCA17) involves the expression of a polyglutamine (polyQ) expanded TATA-binding protein (TBP), a general transcription initiation factor. TBP interacts with other protein factors, including high mobility group box 1 (HMGB1), to regulate gene expression. Previously, our proteomic analysis of soluble proteins prepared from mutant TBP (TBP/Q_61_) expressing cells revealed a reduced concentration of HMGB1. Here, we show that HMGB1 can be incorporated into mutant TBP aggregates, which leads to reduced soluble HMGB1 levels in TBP/Q_61∼79_ expressing cells. HMGB1 overexpression reduced mutant TBP aggregation. HMGB1 cDNA and siRNA co-transfection, as well as an HSPA5 immunoblot and luciferase reporter assay demonstrated the important role of HMGB1 in the regulation of *HSPA5* transcription. In starvation-stressed TBP/Q_36_ and TBP/Q_79_ cells, increased reactive oxygen species generation accelerated the cytoplasmic translocation of HMGB1, which accompanied autophagy activation. However, TBP/Q_79_ cells displayed a decrease in autophagy activation as a result of the reduction in the cytoplasmic HMGB1 level. In neuronal SH-SY5Y cells with induced TBP/Q_61∼79_ expression, HMGB1 expression was reduced and accompanied by a significant reduction in the total outgrowth and branches in the TBP/Q_61∼79_ expressing cells compared with the non-induced cells. The decreased soluble HMGB1 and impaired starvation-induced autophagy in cells suggest that HMGB1 may be a critical modulator of polyQ disease pathology and may represent a target for drug development.

## Introduction

The TATA-binding protein (TBP) is a universal basal transcription factor that is involved in the expression of most eukaryotic genes [1]. TBP contains a polymorphic polyglutamine (polyQ) domain in its N-terminus and a DNA-binding domain in its C-terminus. Although the highly conserved C-terminus mediates the transcriptionally relevant interactions that involve TBP, the evolutionarily divergent N-terminal region may also play a role in transcriptional activation at TATA-containing promoters [2], [3]. In humans, the polyQ tract normally contains 25–42 glutamine residues [4]. Expanded alleles that range from 43 to 66 glutamines have been associated with spinocerebellar ataxia 17 (SCA17) [5], [6], a neurodegenerative disorder characterized by ataxia, dystonia, parkinsonism, dementia and seizures [7], [8].

The high mobility group box 1 (HMGB1) is a ubiquitous and highly conserved nuclear protein with a proposed role in the regulation of eukaryotic gene expression [9], [10]. The domain structure of the 215 amino acid HMGB1 protein consists of two DNA-binding boxes and a C-terminal tail [11]. The basic DNA-binding domain interacts with TBP to affect the transcription factor TFIIB-TBP interaction [12]. Furthermore, the acidic C-terminus interacts with the Q-tract in the N-terminus of TBP to increase the affinity of TBP for the TATA element [13].

In addition to its function as a nuclear regulator of transcription, HMGB1 also plays a role as a cytokine-like molecule when it is released into the extracellular space [14], [15]. HMGB1 regulates autophagy under conditions of oxidative stress [16], [17]. HMGB1 has also been shown to exhibit chaperone-like activity and is a potential therapeutic candidate in polyQ disease [18], [19]. In this study, we demonstrated that mutant TBP aggregates sequestered HMGB1 to reduce functional HMGB1, which leads to a reduction in *HSPA5* gene expression. The overexpression of HMGB1 protein reduced the TBP aggregate formation. In starvation-stressed TBP/Q_79_ cells, sequestration of HMGB1 into TBP aggregates led to decreased autophagy activation as a result of the reduced cytoplasmic HMGB1 level.

## Results

### Incorporation of HMGB1 into mutant TBP aggregates in HEK-293T cells

**Figure 1:**
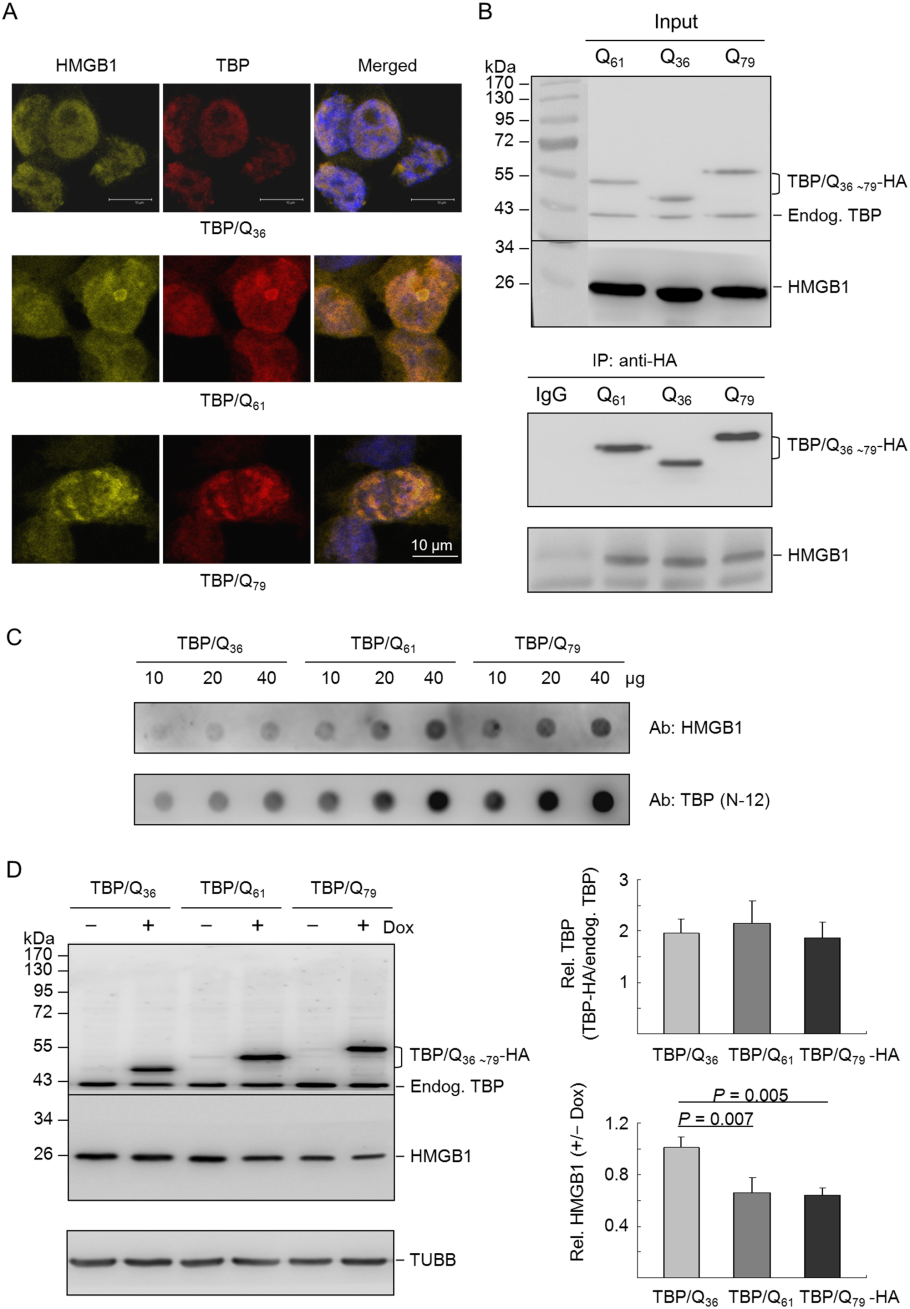
Localization of HMGB1 in TBP/Q_36∼79_-expressing 293 cells. (A) Isogenic 293 cells inducibly expressed TBP/Q_36∼79_ for 6 days. The cells were then fixed and stained with antibodies specific for TBP (red) and endogenous HMGB1 (yellow). The nuclei were counterstained with DAPI (blue). (B) Isogenic 293 cells were induced to express TBP/Q_36∼79_ for 6 days. Cell lysates were prepared (Input, top panel) and immunoprecipitations (IP, bottom panel) were performed with anti-HA antibody. Rabbit IgG was used as a negative control for IP. Cell lysates and immunoprecipitates were immunostained with anti-TBP or anti-HMGB1 antibody. (C) HEK-293T cells were transfected with TBP/Q_36∼79_. After 48 hr, insoluble pellets were collected and lysed in SDS buffer. Lysates (10–40 µg) were filtered through an acetate membrane, and the filter was probed with antibodies to detect the trapped endogenous HMGB1 and transfected TBP. (D) Flp-In isogenic 293 cells were induced (+Dox) for 6 days to express HA-tagged TBP/Q_36∼79_. Cell lysates were prepared and analyzed using anti-TBP and anti-HMGB1 antibodies. The induced TBP/Q_36∼79_-HA protein level relative to endogenous TBP and the level of HMGB1 (+/–Dox) were normalized to the loading control, β-tubulin (TUBB). Data are expressed as the mean ± SD from three independent experiments.

A previous proteomic analysis of soluble nuclear proteins from neurons expressing mutant huntingtin or ataxin-1 revealed reduced HMGB1/2 protein levels [20]. Our proteomics study of isogenic 293 cells expressing TBP/Q_36∼61_ also revealed a 1.48-fold reduction in HMGB1 expression (data not shown). To examine whether HMGB1 incorporates itself into mutant TBP aggregates, 293-derived cells with inducible TBP/Q_36∼79_ expression were examined for endogenous HMGB1 expression after 6 days, positive nuclei with punctuate inclusion bodies that co-localized with HMGB1 were visible in the TBP/Q_61∼79_ cells (Fig. 1A).

To assess the intracellular association of HMGB1 with TBP, the above isogenic 293 cells expressing TBP/Q_36∼79_ were used for a half-*in* *vivo* co-immunoprecipitation assay. The expression of TBP protein was initially examined by Western blotting using a TBP antibody. As shown in the top panel of Fig. 1B, the TBP antibody detected 47 kDa TBP/Q_36_-HA, 50 kDa TBP/Q_61_-HA and 53 kDa TBP/Q_79_-HA proteins in induced cells, in addition to the endogenous 43 kDa TBP protein. The cell lysates were then subjected to immunoprecipitation with anti-HA antibody. As shown in the bottom panel of Fig. 1B, immunoblotting analysis showed that induced TBP proteins were co-immunoprecipitated with HMGB1. When the TBP/Q_36∼79_ transfected samples were subjected to a filter trap assay and stained with an anti-HMGB1 or anti-TBP antibody, the incorporation of HMGB1 into the SDS-insoluble aggregates with TBP/Q_61_ and TBP/Q_79_ was also clearly increased (Fig. 1C).

To test whether TBP/Q_61∼79_ expression suppressed the level of soluble HMGB1 protein, the expressed TBP protein levels were examined by Western blot analysis using anti-TBP antibody after 6 days of induction with doxycycline (+Dox) or not (−Dox). As shown in Fig. 1D, in addition to the 43 kDa endogenous TBP protein, the TBP antibody detected 47∼53 kDa HA-tagged TBP/Q_36∼79_ proteins in the doxycycline-induced TBP cells (187–216% of the endogenous TBP). According to HMGB1 antibody staining, the cells that expressed HA-tagged TBP/Q_36_, TBP/Q_61_ and TBP/Q_79_ displayed 100.7%, 66.0%, and 64.1%, respectively, of soluble HMGB1 protein compared with the non-induced cells. Thus, a significant reduction in endogenous soluble HMGB1 protein was observed in the induced TBP/Q_61∼79_ cells compared with the induced TBP/Q_36_ cells (66.0–64.1% vs. 100.7%, respectively, *P* = 0.007–0.005).

### HMGB1 overexpression reduces TBP aggregation

**Figure 2:**
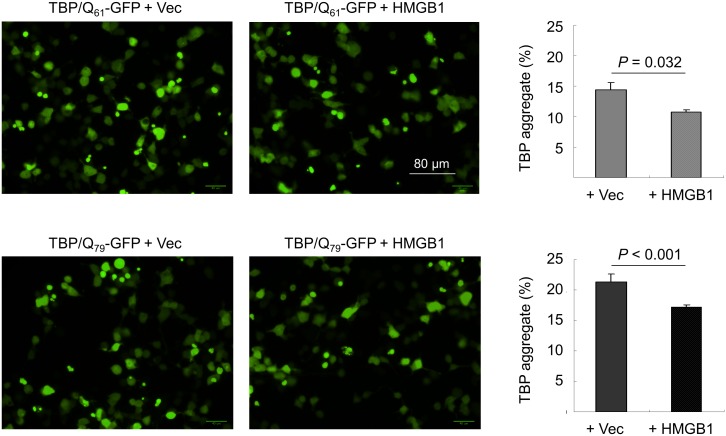
Overexpression of HMGB1 in the SCA17 transient cell model. HEK-293T cells were co-transfected with plasmids encoding TBP/Q_61∼79_-GFP and pcDNA3 without (Vec) or with (+HMGB1) *HMGB1* cDNA. After 2 days, the cells were examined directly using fluorescence microscopy. The percent aggregation was assessed using the HCA system with excitation/emission wavelengths of 482/536 nm for GFP.

To determine whether HMGB1 could suppress the aggregation of mutant TBP, we transiently co-expressed HMGB1 with N-terminal TBP/Q_61∼79_-GFP in HEK-293T cells. Fig. 2 shows the fluorescence microscopy images. Without HMGB1 co-transfection, 14.3–21.4% of the TBP/Q_61∼79_-GFP cells developed visible aggregates. Co-transfection with HMGB1 effectively reduced the number of cells with aggregates to 10.8% (*P* = 0.032) and 17.2% (*P*<0.001) in the TBP/Q_61_-GFP and TBP/Q_79_-GFP cells, respectively.

### HMGB1 regulation of *HSPA5* transcription in TBP/Q_61_ expressing cells

**Figure 3:**
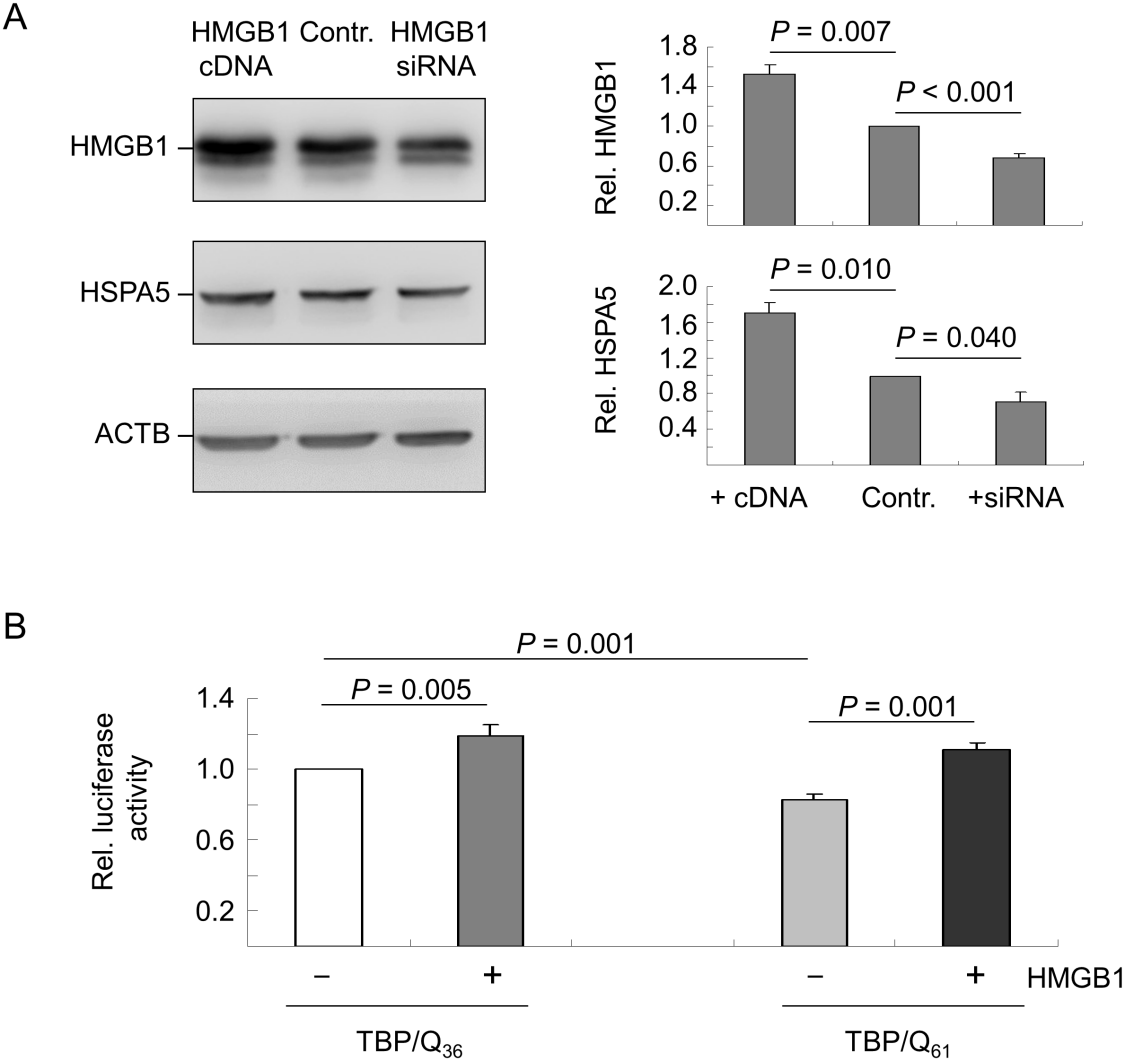
HMGB1 modulation of *HSPA5* gene transcription. (A) HEK-293T cells were transiently transfected with *HMGB1* cDNA or siRNA. After 2 days, the cell lysates were prepared and analyzed using anti-HMGB1 and anti-HSPA5 antibodies. The HMGB1 and HSPA5 protein levels were normalized to the loading control, β-actin (ACTB). The levels of HMGB1 and HSPA5 proteins in the vector-transfected cells (Contr.) were set to 100%. The data are expressed as the mean ± SD values from three independent experiments. (B) 293-derived TBP/Q_36∼61_ cells were induced for 2 days to express TBP. The *HSPA5* luciferase reporter and pcDNA3 without (–) or with (+) *HMGB1* cDNA were then transiently expressed for an additional 2 days. The levels of luciferase activity were expressed as fold change compared with the activity of the *HSPA5* reporter in cells expressing TBP/Q_36_. Each value represents the mean ± SD of three independent experiments; each experiment was performed in duplicate.

Previously, our protein studies on human cells expressing polyQ expanded TBP revealed a reduced expression level of HSPA5 [21], [22]. To examine whether the reduced level of soluble HMGB1 attenuated *HSPA5* expression, *HMGB1* cDNA and siRNA were prepared and tested in HEK-293T cell transfection assays. Immunoblot analysis was first performed to assess the expression levels of HMGB1. As shown in Fig. 3A, HMGB1 was overexpressed at 146% (*P* = 0.007) and depleted to 69% (*P*<0.001) in the cDNA and siRNA-transfected cells, respectively. We then examined the expression levels of HSPA5 in the cells transfected with *HMGB1* cDNA or siRNA. As shown in Fig. 3A, the cells transfected with *HMGB1* cDNA exhibited 170% (*P* = 0.010), whereas the cells transfected with *HMGB1* siRNA exhibited 71% (*P* = 0.040) compared with the respective control. Thus, the results suggest that HMGB1 could modulate *HSPA5* gene expression.

To further evaluate the HMGB1-TBP interaction in the regulation of *HSPA5* gene expression, we used a luciferase reporter assay to assess the expression of HSPA5 in cells that expressed normal TBP/Q_36_ or expanded TBP/Q_61_ with or without transient HMGB1 overexpression. As shown in Fig. 3B, when the luciferase activity for the *HSPA5* reporter in TBP/Q_36_ cells was maintained at 100%, the luciferase activities driven by the *HSPA5* promoter in the TBP/Q_36_ cells with HMGB1 overexpression, in the TBP/Q_61_ cells without HMGB1 overexpression and in the TBP/Q_61_ cells with HMGB1 overexpression were 119%, 83%, and 111%, respectively. Thus, the *HSPA5* luciferase reporter was activated in cells that overexpressed HMGB1 (TBP/Q_36_ cells: 119% vs. 100%, *P* = 0.005; TBP/Q_61_ cells: 111% vs. 83%, *P* = 0.001). While a significant difference was observed between the TBP/Q_36_ and TBP/Q_61_ cells without HMGB1 overexpression (100% vs. 83%, respectively, *P* = 0.001), there was no significant difference between the TBP/Q_36_ and TBP/Q_61_ cells with HMGB1 overexpression (119% vs. 111%, respectively, *P* = 0.123).

### Oxidative stress and nuclear and cytoplasmic HMGB1 levels in TBP/Q_79_ expressing cells

**Figure 4:**
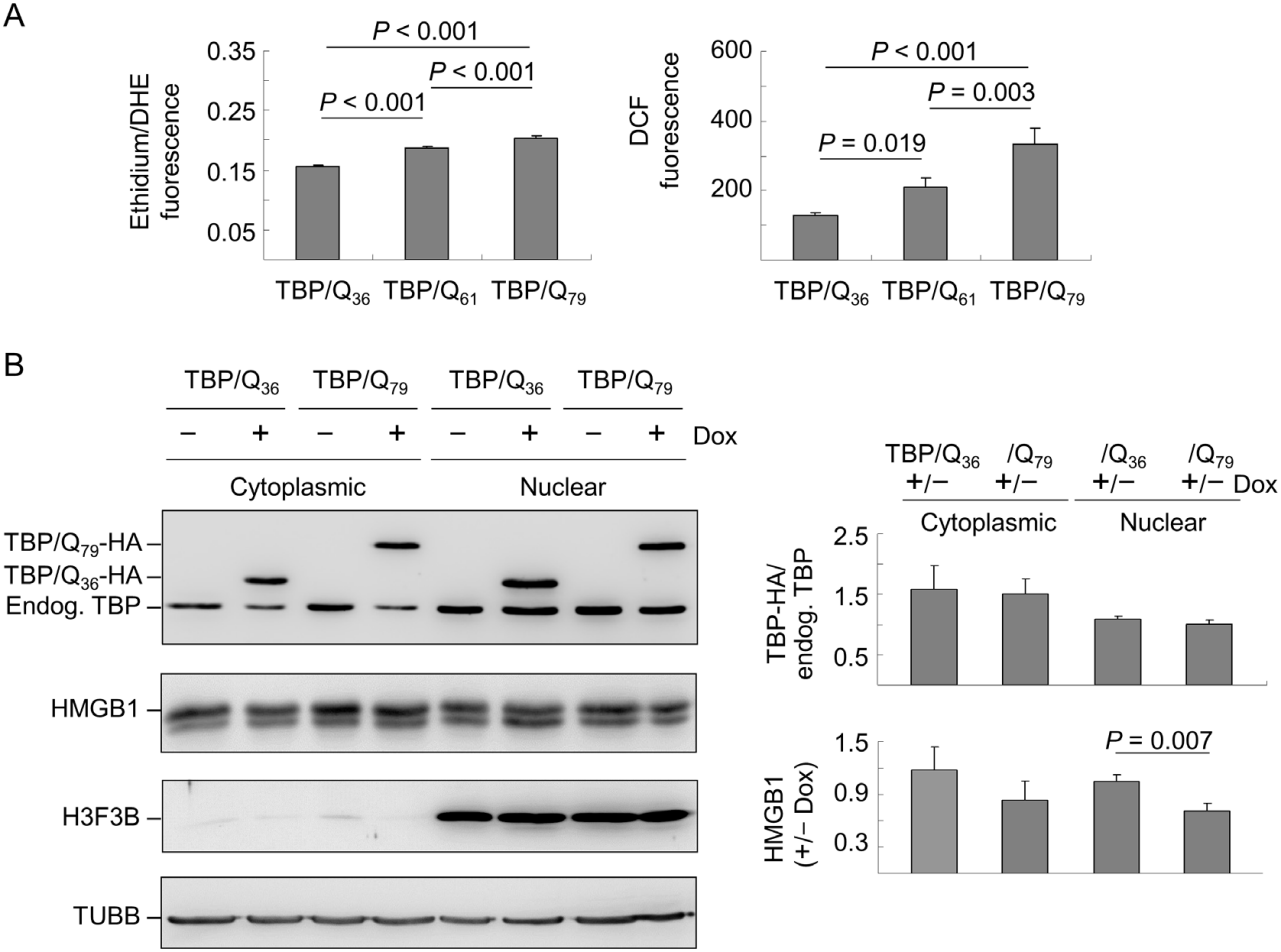
ROS and HMGB1 distribution in TBP/Q_36∼79_ 293 cells. (A) 293-derived cells were induced for 6 days to express TBP/Q_36∼79_. The cells were loaded with DHE and DCFH-DA, and superoxide/peroxide production was fluorometrically quantified. The data are expressed as the mean ± SD of three independent experiments. (B) Cells were induced for 6 days to express TBP/Q_36_ and TBP/Q_79_. The extracts were fractionated into cytoplasmic and nuclear components and analyzed using TBP, HMGB1, H3F3B (histone H3.3) and TUBB antibodies. The induced TBP/Q_36∼79_-HA protein level relative to endogenous TBP and the level of HMGB1 (+/–Dox) were normalized to the loading control, β-tubulin (TUBB). Values are expressed as the means ± SD of three independent experiments.

In addition to its functions as an architectural chromatin binding factor and a nuclear regulator of transcription, HMGB1 is also a critical regulator of autophagy [16]. It has been shown that mutant Htt caused increased levels of ROS in neuronal and non-neuronal cells [23], and HMGB1 regulated autophag**y** in response to this oxidative stress [17]. By producing ROS, mutant TBP may induce oxidative stress, which results in the relocation of HMGB1 from the nucleus to the cytoplasm to activate an autophagic response to oxidative stress. To test this hypothesis, we induced the expression of TBP/Q_36∼79_ in 293 cells and used two oxidation-sensitive compounds, dihydroethidium (DHE) and dichlorofluorescein diacetate (DCFH-DA), to measure intracellular ROS. As shown in Fig. 4A, superoxide production in TBP/Q_61_ and TBP/Q_79_ cells was significantly increased compared with TBP/Q_36_ cells (ethidium/DHE fluorescence 0.186–0.204 vs. 0.156, *P*<0.001). This remained true for peroxide production (DCF fluorescence 210–333 vs. 127, *P* = 0.019-<0.001). The superoxide/peroxide production between TBP/Q_61_ and TBP/Q_79_ cells was also significantly different (*P*<0.001 for ethidium/DHE fluorescence and *P* = 0.003 for DCF fluorescence). These results suggested that ROS generation was polyQ length-dependent in TBP/Q_61∼79_ expressing cells.

We then examined the HMGB1 levels in the nucleus and cytoplasm. As demonstrated by Western blot analysis (Fig. 4B), with adding doxycycline to induce HA-tagged TBP expression, TBP/Q_36_-HA was expressed at 158.0% and TBP/Q_79_-HA was expressed at 150.1% in the cytoplasmic fraction (*P* = 0.788) and TBP/Q_36_-HA expression was 108.8% and TBP/Q_79_-HA was 101.1% in the nuclear fraction (*P* = 0.175) compared with endogenous TBP. After HMGB1 antibody immunostaining, 117.9% of cytoplasmic and 104.3% of nuclear HMGB1 were observed in induced TBP/Q_36_ cells and 83.2% of cytoplasmic and 71.4% of nuclear HMGB1 were observed in induced TBP/Q_79_ cells compared with the non-induced cells. The cytoplasmic HMGB1 protein tended to decrease in the induced TBP/Q_79_ cells compared with the induced TBP/Q_36_ cells (117.9% vs. 83.2%, respectively, *P* = 0.153). A significant reduction in nuclear HMGB1 protein was observed in the induced TBP/Q_79_ cells compared with the induced TBP/Q_36_ cells (104.3% vs. 71.4%, respectively, *P* = 0.007).

### Reduced HMGB1 level and autophagy activation in starvation-stressed TBP/Q_79_ expressing cells

**Figure 5:**
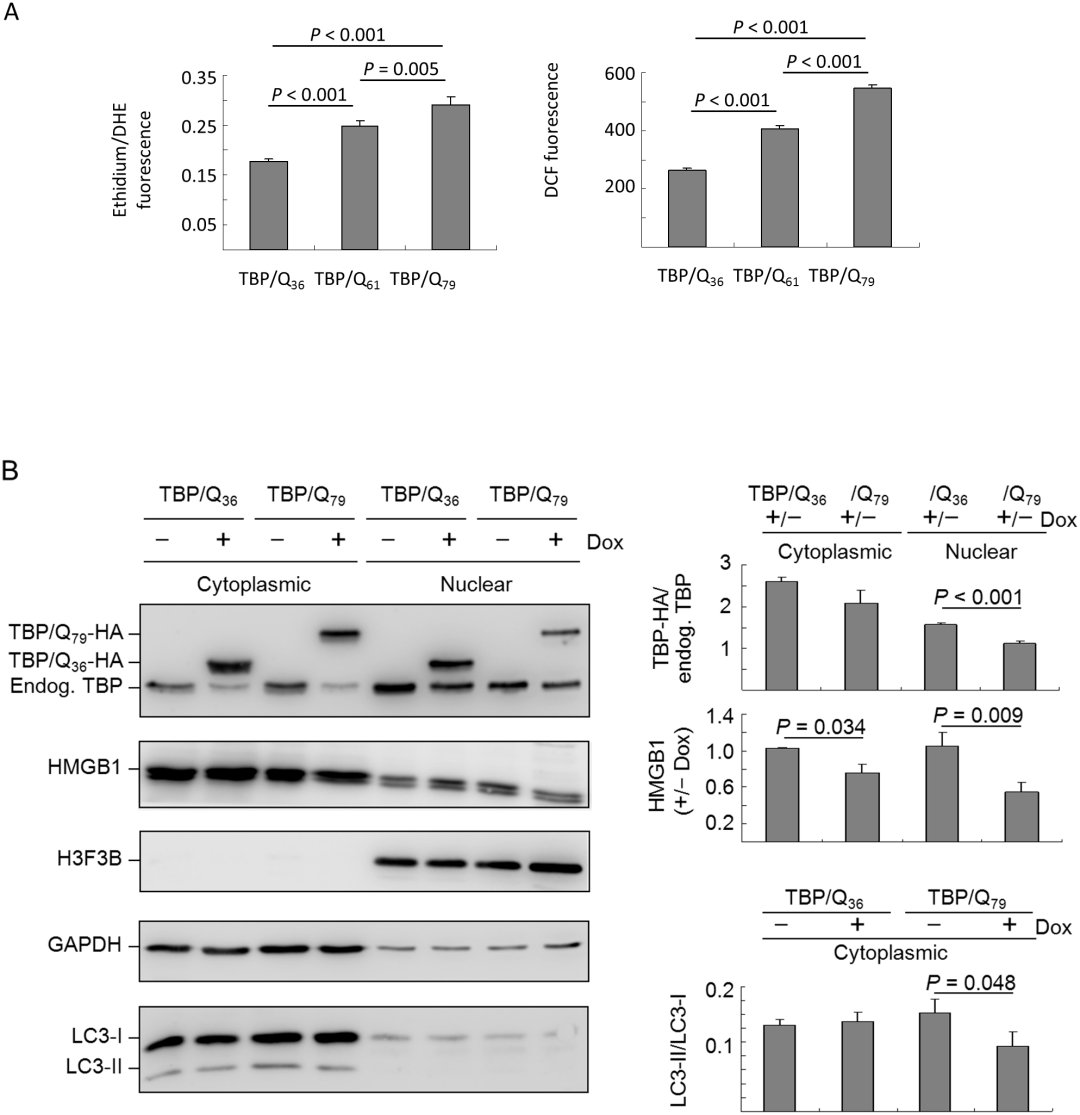
ROS and HMGB1 distribution in starvation-stressed TBP/Q_36∼79_ 293 cells. (A) 293-derived cells were induced for 4 days to express TBP/Q_36∼79_. The cells were then placed in medium containing 0.5% fetal bovine serum for 2 additional days. The cells were loaded with DHE and DCFH-DA, and superoxide/peroxide production was fluorometrically quantified. The data are expressed as the mean ± SD of three independent experiments. (B) Cells were induced for 6 days to express TBP/Q_36_ and TBP/Q_79_, with starvation-stress in the last two days. The extracts were fractionated into the cytoplasmic and nuclear components and analyzed using TBP, HMGB1, H3F3B, GAPDH and LC3 antibodies.

Autophagy is traditionally associated with oxidative stress. ROS are signaling molecules that are critical for the activation of nutrient starvation-induced autophagy [24]. We examined ROS production, HMGB1 level and autophagy activation in TBP/Q_79_ expressing cells following 48 hr serum starvation. As shown in Fig. 5A, significantly increased levels of superoxide and peroxide production were observed in the TBP/Q_61∼79_ cells compared with the TBP/Q_36_ cells (ethidium/DHE fluorescence 0.247–0.291 vs. 0.178, respectively, *P*<0.001; DCF fluorescence 407–545 vs. 265, respectively, *P*<0.001) in addition to the significantly increased levels of superoxide/peroxide production observed between the TBP/Q_61_ and TBP/Q_79_ cells (*P* = 0.005 for ethidium/DHE fluorescence and *P*<0.001 for DCF fluorescence). Significantly increased levels of superoxide/peroxide were observed in the starvation-stressed cells compared with the non-stressed cells expressing TBP/Q_36_ (*P* = 0.002-<0.001), TBP/Q_61_ (*P* = 0.001-<0.001) or TBP/Q_79_ (*P* = 0.002–0.001).

Western blot experiments were then performed to examine starvation associated with reduced nuclear or cytoplasmic HMGB1 level (Fig. 5B). Immunocytochemical staining with the TBP antibody demonstrated that TBP/Q_36_-HA expression was 261.7% and TBP/Q_79_-HA expression was 207.9% in the cytoplasmic fraction (*P* = 0.090), and TBP/Q_36_-HA expression was 156.2% and TBP/Q_79_-HA was expressed at 112.0% in the nuclear fraction (*P*<0.001) compared with the endogenous TBP. Using the HMGB1 antibody, 102.5% cytoplasmic and 105.6% nuclear HMGB1 were observed in the induced TBP/Q_36_ cells, and 76.1% cytoplasmic and 54.6% nuclear HMGB1 were observed in the induced TBP/Q_79_ cells compared with the non-induced cells. Significant reductions in the cytoplasmic and nuclear levels of HMGB1 were observed in the induced TBP/Q_79_ cells compared with the induced TBP/Q_36_ cells (cytoplasmic: 102.5% vs. 76.1%, respectively, *P* = 0.034; nuclear: 105.6% vs. 54.6%, respectively, *P* = 0.009).

Autophagy activation was examined in starvation-stressed TBP/Q_79_ expressing cells. The expression levels of lipid phosphatidylethanolamine-conjugated LC3-II were compared with the levels of cytosolic LC3-I in the cytoplasmic fractions of TBP/Q_36_ and TBP/Q_79_ cells with or without doxycycline treatment because LC3-II is the only known protein that specifically associates with autophagosomes and not with other vesicular structures [25]. As shown in Fig. 5B, the starvation-induced expression of TBP/Q_79_ significantly reduced the LC3-II/LC3-I ratio (9.5% vs. 14.2%, respectively, *P* = 0.048), whereas the starvation-induced expression of TBP/Q_36_ did not affect the LC3-II/LC3-I ratio under the same conditions (13.0% vs. 12.4%, respectively, *P* = 0.606). Thus, these results suggest that cytoplasmic HMGB1 level and autophagy activation were reduced in starvation-stressed TBP/Q_79_ expressing cells.

### Reduced HMGB1 in TBP/Q_79_ expressing SH-SY5Y cells and the associated neuronal phenotype

**Figure 6:**
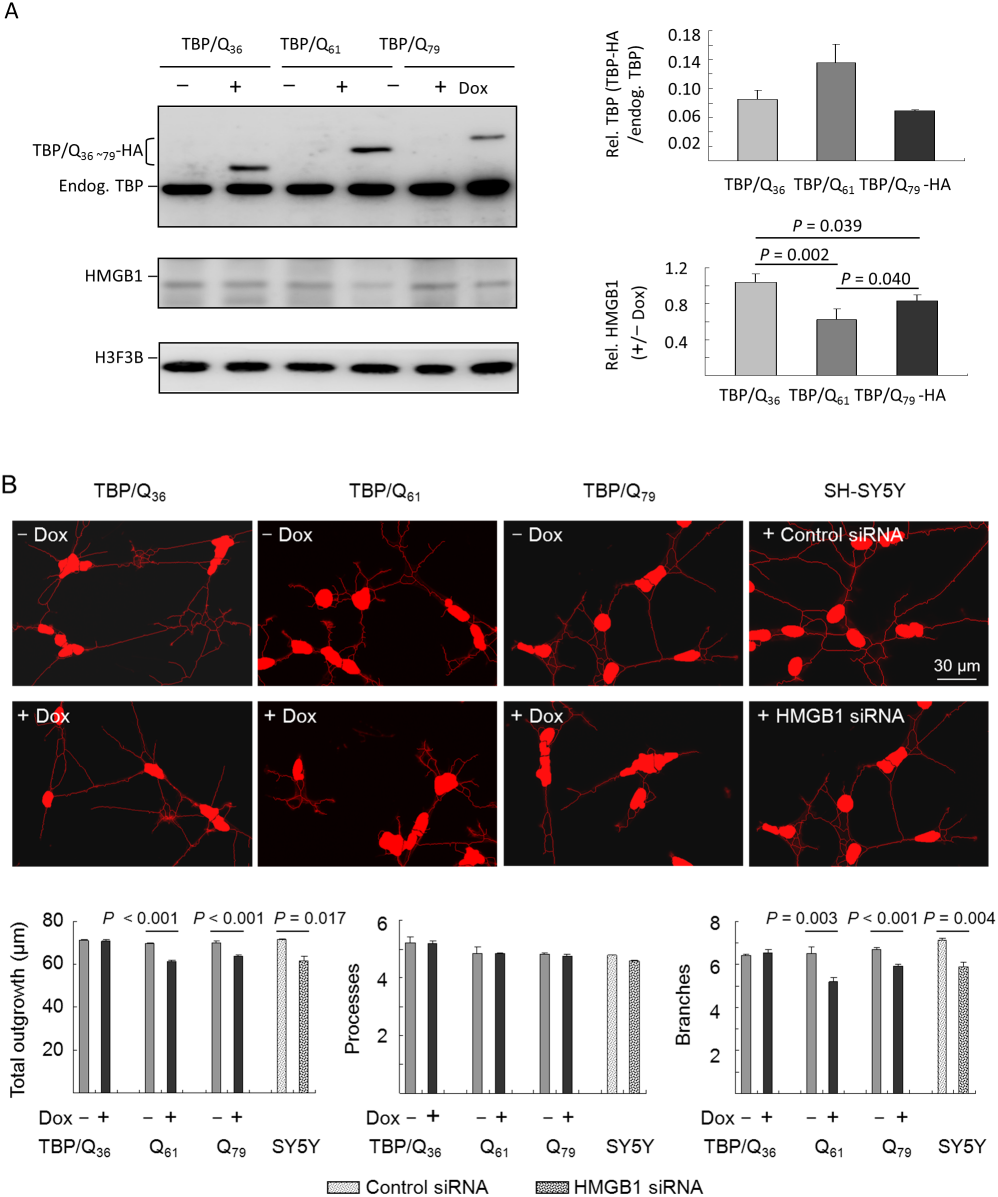
HMGB1 dysfunction in TBP/Q_61∼79_ SH-SY5Y cells and the associated neuronal phenotype. (A) SH-SY5Y-derived TBP/Q_36∼79_ cells were induced for 6 days to express TBP. The cell lysates were prepared and analyzed using anti-TBP and anti-HMGB1 antibodies. The induced TBP/Q_36∼79_-HA protein level relative to endogenous TBP and the level of HMGB1 (+/–Dox) were normalized to the loading control, H3F3B. The data are expressed as the mean ± SD values from three independent experiments. (B) Differentiation was induced in the SH-SY5Y-derived TBP/Q_36∼79_ cells using *trans* retinoic acid. The next day, doxycycline was added to induce TBP/Q_36∼79_ expression for 10 days (+Dox). For HMGB1 knockdown, siRNA was electroporated into the SH-SY5Y cells, and differentiation was then induced using *trans* retinoic acid for 10 days. The cells were subsequently stained with phalloidin, and morphologic differentiation was assessed by quantifying neuronal outgrowth, processes and branches.

The SH-SY5Y-derived isogenic cells with inducible TBP/Q_36∼79_ expression were used to examine whether the expression of TBP/Q_61∼79_ suppressed the level of soluble HMGB1 protein in neuronal cells. After six days of induction, TBP and HMGB1 levels were examined by Western blot using TBP and HMGB1 antibodies. Although the levels of the induced TBP/Q_36∼79_-HA proteins were low (6.9–14.6%) compared with endogenous TBP (Fig. 6A), a significant reduction in the endogenous soluble HMGB1 protein was observed in the SH-SY5Y cells expressing TBP/Q_61∼79_-HA compared with the induced TBP/Q_36_ cells (62.2–83.0% vs. 100%, respectively, *P* = 0.002–0.039). When TBP/Q_61∼79_ SH-SY5Y cells were differentiated by incubation in retinoic acid for ten days, significant reductions in the total outgrowth and branching were observed compared with the non-induced cells (61 µm vs. 69.4 µm, respectively, in the TBP/Q_61_ cells (*P*<0.001) and 63.7 µm vs. 70.1 µm, respectively, in the TBP/Q_79_ cells (*P*<0.001) for total outgrowth; 5.2 vs. 6.5, respectively, in the TBP/Q_61_ cells (*P* = 0.003) and 5.9 vs. 6.7, respectively, in the TBP/Q_79_ cells (*P*<0.001) for branching (Fig. 6B)). Depletion of HMGB1 by siRNA (50%, data not shown) also significantly reduced the total outgrowth (61.6 µm vs. 71.6 µm, respectively, *P* = 0.017) and branching (5.9 vs. 7.1, respectively, *P* = 0.004) of SH-SY5Y cells (Fig. 6B). Nevertheless, no significant reduction of process was observed between non-induced and induced TBP/Q_61∼79_ cells (4.8_∼_4.9 vs. 4.7_∼_4.8) or between siRNA untreated and treated (4.8 vs. 4.6) cells (*P*>0.05).

## Discussion

Transcriptional dysfunction is a key feature of polyQ disease pathology [26]. Mutant polyQ proteins may impair the functions of nuclear factors through altered interactions with nuclear factors or sequestration into polyQ aggregates, which leads to transcriptional dysfunction. It has been reported that mutant TBP enhanced the interaction of TBP with the general transcription factor IIB and transcription factor nuclear factor Y (NFY), thereby inhibiting their associations with the promoters of the *Hsp25*, *Hsp70* and/or *HspA5* genes in SCA17 mouse models [27], [28]. Our SCA17 cellular model studies have also revealed NFY dysfunction and reduced expression of HSPB1, HSPA8 and HSPA5 [21], [22], [29]. Because reduced HMGB1 protein was observed in the soluble fraction of cells expressing mutant TBP, we examined the role of HMGB1 in SCA17 pathogenesis. We determined that HMGB1 was incorporated into mutant TBP aggregates, which led to reduced levels of soluble HMGB1 in TBP/Q_61∼79_ expressing cells (Fig. 1). This result is consistent with the previously reported reduction in the concentration of soluble HMGB1/2 proteins in neurons expressing huntingtin or ataxin-1 [20]. HMGB1 overexpression also reduced mutant TBP aggregation in cells (Fig. 2), which is consistent with the observation that compensatory expression of the HMGB proteins ameliorated polyQ-induced pathologies in primary neurons and *Drosophila* polyQ models [20].

In addition to its role as a nuclear architectural factor and a secreted inflammatory factor, HMGB1 has been reported to function in the regulation of gene expression [9], [10], [12]. Through its binding to the polyQ tract of TBP [13], HMGB1 is a likely participant in the pathogenesis of SCA17. Using *HMGB1* cDNA and siRNA co-transfection and HSPA5 immunoblot and reporter assays, we determined that HMGB1 could function as a nuclear factor to regulate *HSPA5* transcription, and reduced soluble HMGB1 in the nucleus attenuates *HSPA5* promoter activity in TBP/Q_61_ expressing cells, which can be compensated by increased HMGB1 expression (Fig. 3). HSPA5 (also known as 78 kDa glucose-regulated heat shock protein, GRP-78) is involved in the folding and assembly of proteins in the endoplasmic reticulum. Decreased *HSPA5* transcription in TBP/Q_61_ cells may lead to impaired protein folding, a reduction in the stress response, and the induction of cell death. Because complete elimination of HSPA5 in Purkinje cells leads to reduced cytosolic ubiquitination and accelerated cerebellar degeneration in a mouse model [30], HMGB1 could be a target of mutant TBP in SCA17, as well as NFYA [29].

The mechanism of HMGB1-modulated *HSPA5* expression is not clear. Previously, we demonstrated that mutant TBP aggregates sequester NFYA, which leads to a reduction in *HSPA5* transcription in SCA17 [29]. NFYA binds to CCAAT motifs in the promoter regions of a variety of genes [31], [32], including *HSPA5*. Despite incorporation into mutant TBP aggregates, HMGB1 may not regulate *HSPA5* transcription directly, as previously discussed. Because HMGB1/2 modulates the expression of human topo IIα by enhancing NFY binding to the topo IIα promoter [33], HMGB1 may increase HSPA5 expression by enhancing NFY binding to the *HSPA5* promoter. However, this hypothesis requires additional studies to confirm this theory.

Autophagy is a cellular pathway involved in protein and organelle degradation and is primarily a protective process for cell survival [34]. Autophagy has been reported to be activated in a diverse set of pathologies, including infections, cancer, neurodegeneration, heart disease and nutrient deprivation [35]. In polyQ diseases, autophagy has been proposed as a strategy to reduce the accumulation of mutant polyQ aggregates and protect against mutant protein neurotoxicity [19]. HMGB1 is a critical regulator of autophagy [16], and the inhibition of HMGB1 release or the loss of HMGB1 decreases the formation of autophagolysosomes under oxidative stress [17]. In our starvation-stressed TBP/Q_79_ expressing cells, autophagy dysfunction was observed, as autophagy activation decreased along with a reduction in relocated cytoplasmic HMGB1 (Fig. 5). These results, along with reports that autophagy induction reduces mutant ataxin-3 levels and toxicity in a SCA3 mouse model [36], suggest that targeting cytoplasmic HMGB1 to increase the clearance of mutant polyQ proteins may provide a potential therapeutic approach for SCA17.

The SH-SY5Y neuronal model with inducible TBP/Q_36∼79_ expression was established to study SCA17 pathogenesis. Although the protein cannot be induced at very high levels (Fig. 6), SH-SY5Y cells expressing N-terminal TBP/Q_36∼79_ at a level several fold higher than endogenous TBP expression can be established (data not shown). For unknown reasons, full-length TBP in neuronal cell lines cannot be over-induced. Similar to TBP, most polyQ disease proteins are widely expressed, and some proteins are involved in critical cellular processes. However, the underlying mechanisms for the selective neurodegeneration in distinct brain regions remain paradoxical. How polyQ tract expansions mediate the patterns of neuronal cell loss observed in each disease is not completely unclear. Nevertheless, the mechanisms proposed thus far provide explanations for the acceleration of neuronal dysfunction and/or cell death in specific neurons. We will continue to improve our SCA17 neuronal models to assess potential therapeutic targets underlying SCA17 pathogenesis.

SCA17 is an autosomal dominant disease. Most sufferers are heterozygous for the pathogenic TBP mutation. As the promoter strength of both alleles is compatible, it is expected that the expression levels of normal and pathogenic TBP are equal in patients. Nevertheless, the quantity of mutant protein in patients showed different expression levels, at most the mutant allele has slightly decreased expression level compared to the normal allele [22]. In our cultured cell models, the expression levels of exogenous TBP are roughly 0.5–1.5 folds of those of endogenous TBP, which recapitulates the condition of SCA17 patients. Additionally, the expression of exogenous TBP did not reduce significantly the expression of endogenous TBP in most cases (i.e. Figs. 1D and 6A), although we did observe in some experiments that expression of exogenous TBP reduced the expression of endogenous TBP (Figs. 4B and 5B). In the latter cases, the amount of reduced endogenous TBP is compatible in both cells expressing TBP/Q_36_ and TBP/Q_79_, indicating that the reduction of endogenous TBP might not be a prominent factor contributing to the pathological changes in these SCA17 cellular models. In contrast, the reduction of HMGB1 is consistently observed in cells expressing pathogenic TBP (Figs. 1D, 4B, 5B and 6A). Therefore, HMGB1 may be a critical modulator of SCA17 pathogenesis and may represent a target for drug development.

It is of note that most of the observed effects are quite modest, although statistically significant in the cell models over-expressing polyQ-expanded TBP. The reason for that is proposed to be twofold. First, the magnitude of induction in the cells over-expressing polyQ-expanded TBP (around two times of endogenous TBP) is small, which may lead to modest effects observed in this study. Second, there are probably other pathways involved in the pathogenesis, not yet identified in these cell models. Similar to other polyQ diseases, several pathogenic pathways contribute to neuronal loss, although the effect of each pathway may be small [37]. Therefore, although the observed effects are not great, they may be still authentic, in terms of their contribution to the pathogenesis of SCA17.

In summary, we demonstrated that mutant TBP sequestered HMGB1 into polyQ aggregates, which led to nuclear *HSPA5* transcriptional dysfunction. Under increasing oxidation stress caused by nutrient deprivation, reduced autophagy activation may worsen the pathological process of SCA17. Because increased expression of HMGB1 could effectively suppress mutant TBP aggregation, HMGB1 may be a critical regulator of pathogenesis and a potential therapeutic target for SCA17. Further studies are warranted to confirm the significance of this speculation.

## Materials and Methods

### TBP/Q_36∼79_ cDNA constructs

The HA-tagged full-length TBP/Q_36∼79_ cDNAs in the pGEM-T Easy (Promega) and pcDNA5/FRT/TO (Invitrogen) vectors were generated as previously described [21], [29]. For the construction of TBP/Q_61_-GFP, the *Eco*RI (in the MCS of pGEM-T Easy)-*Rsa*I fragment containing N-terminal TBP/Q_61_ (185 amino acids) was removed from the cloned TBP/Q_61_ cDNA and fused in-frame to the GFP gene in the pEGFP-N1 vector (between the *Eco*RI and *Rsa*I sites in the MCS) (Clontech). The Kozak sequence of the GFP gene was removed by PCR using the site-directed primer CGGGCCCGGGATCCACCGGTCGCCΔGTGAGCAAGGGCGAGGAGCTG (Δ = ACCATG); the deleted Kozak sequence was confirmed by DNA sequencing.

### *HMGB1* cDNA cloning

Polyadenylated RNA (200 ng) isolated from neuroblastoma SK-N-SH cells (ATCC No. HTB11) was reverse transcribed using SuperScript III reverse transcriptase (Invitrogen). The sense and antisense primers used for *HMGB1* cDNA amplification were 5′ TCGCTGAGGAAAAACAACT and 5′ AAAACTGCGCTAGAACCAACTTAT, respectively. The underlined base in the 5′ primer was used to remove the in-frame stop codon in the 5′ untranslated region. The amplified 691-bp *HMGB1* cDNA was cloned into pGEM-T Easy and sequenced. The cDNA was then excised with *Eco*RI and subcloned into pcDNA3 (Invitrogen) for transient expression studies.

### Cell culture and transfection

HEK-293T cells (ATCC No. CRL-11268) were cultured in Dulbecco’s modified Eagle’s medium containing 10% fetal bovine serum (FBS) in a 37°C humidified incubator with a 5% CO_2_ atmosphere. HEK-293-derived cells that inducibly expressed TBP/Q_36∼79_ were constructed as previously described [21] and maintained in medium containing 5 µg/ml blasticidin and 100 µg/ml hygromycin. Doxycycline (5 µg/ml) was used to induce TBP expression. For transient expression, the cells were plated in 6-well (7×10^5^/well) or 12-well (on coverslips, 2×10^5^/well) dishes, grown for 20 hr, and transfected with TBP/Q_36∼79_ and/or *HMGB1* cDNA plasmids (6 µg each/6-well or 2 µg each/12-well) using the T-Pro reagent (JF Biotechnology, Taiwan). siRNA specifically targeting *HMGB1* (100 nM SASI_Hs01_00196036, Sigma) was used to deplete cells of HMGB1. The cells were grown for 48 hr prior to immunocytochemical staining (12-well dishes) and dot/Western blot studies (6-well dishes).

### Immunocytochemical staining

The cells were washed with phosphate-buffered saline (PBS) and fixed in 4% paraformaldehyde in PBS for 10 min, followed by a 20 min incubation with 0.1% Triton X-100 in PBS to permeate the cells; an overnight incubation with 0.5% bovine serum albumin (BSA) in PBS and 20 mM glycine was used to block non-specific binding. Primary antibodies against TBP (N-12, Santa Cruz) and HMGB1 (Abcam), diluted 1∶100 in 1% BSA in PBS, were used to the stain cells at 4°C overnight. After washing, the cells were incubated for 2 hr at room temperature in fluorescein-conjugated secondary antibody (Zymax) diluted 1∶500 in PBS containing 1% BSA and washed with PBS. The nuclei were counterstained using 4′-6-diamidino-2-phenylindole (DAPI) (0.1 µg/ml, Sigma). The stained cells were examined for dual fluorescent imaging using a Leica TCS confocal laser scanning microscope.

### Immunoprecipitation

Total soluble protein from HEK-293-derived cells that inducibly expressed TBP/Q_36∼79_ for 6 days was prepared using buffer containing 50 mM Tris-HCl, 150 mM NaCl, 1 mM EDTA, 1 mM EGTA, 0.1% SDS, 0.5% sodium deoxycholate, 1% Triton X-100, and a protease inhibitor cocktail (Calbiochem). After sonication and centrifugation at 15,000 g for 10 min, the protein concentration was determined (Bio-Rad Protein Assay) using BSA as a standard. Proteins were incubated with anti-HA (Sigma) or rabbit IgG (4 µg per 200 µg of total proteins in 300 µl reaction) at 4°C with continuous mixing overnight. Next day the antibody-protein complex was captured with Protein G Magnetic Beads (30 µl, GE Healthcare) and analyzed by Western blotting as described below.

### Western blot analysis

Soluble proteins (25 µg) prepared as described in immunoprecipitation were separated by 10% SDS-polyacrylamide gel electrophoresis and blotted onto nitrocellulose membranes by reverse electrophoresis. After blocking, the membrane was stained with antibodies against TBP (1TBP18) (1∶3000 dilution, GeneTex), HMGB1 (1∶2000 dilution, Sigma), HSPA5 (1∶500 dilution, Santa Cruz), LC3 (1∶1000 dilution, Novus), H3F3B (1∶3000 dilution, GeneTex), ACTB (1∶5000 dilution, Novus), TUBB (1∶5000 dilution, Sigma) or GAPDH (1∶1000 dilution, MDBio). The immune complexes were detected using horseradish peroxidase-conjugated goat anti-mouse or goat anti-rabbit (Jackson ImmunoResearch) IgG antibody (1∶10000 dilution) and a chemiluminescent substrate (Millipore).

### Dot blot filter retardation assay

Transfected cells were lysed on ice for 30 min in buffer containing 50 mM Tris-HCl pH 8.8, 100 mM NaCl, 5 mM MgCl_2_, 0.5% (w/v) NP40, 100 mM EDTA and a protease inhibitor cocktail. After centrifugation for 5 min at 14,000 rpm, insoluble pellets were resuspended in buffer (20 mM Tris-HCl pH 8.0, 15 mM MgCl_2_) containing DNase I (0.5 mg/ml). After incubation at 37°C for 1 hr, the reaction was terminated by adjusting the mixture to 20 mM EDTA, 2% SDS and 50 mM DTT, followed by heating at 98°C for 5 min. Extracted proteins (10–40 µg) were diluted in 2% SDS and filtered through a cellulose acetate membrane (0.2 µm pore size, Schleicher and Schuell), which was pre-equilibrated with 2% SDS, using a BRL dot-blot filtration unit. The filters were washed twice in 0.1% SDS, blocked in TBS containing 3% nonfat dried milk and stained with the TBP (N-12) (1∶500 dilution, Santa Cruz) or HMGB1 (1∶2000 dilution, Abcam) antibody. The immune complexes on the filter were detected as previously described.

### Fractionation of cytoplasmic and nuclear proteins

Cells were re-suspended in harvest buffer (10 mM HEPES pH 7.9, 50 mM NaC1, 0.5 M sucrose, 0.1 mM EDTA, 0.5% Triton X-100, 1 mM DTT, 10 mM tetrasodium pyrophosphate, 1 mM NaF, and 17.5 mM β-glycerolphosphate) containing a protease inhibitor cocktail for 5 min. The cells were then centrifuged at 1,000×g for 10 min at 4°C to pellet the nuclei. The post nuclear supernatant was centrifuged at 14,000×g for 15 min at 4°C to obtain the cytoplasmic extract. The nuclear pellet was then washed in buffer containing 10 mM HEPES pH 7.9, 10 mM KC1, 0.1 mM EDTA, 0.1 mM EGTA, 1 mM DTT and a protease inhibitor cocktail and re-suspended in buffer containing 10 mM HEPES pH 7.9, 500 mM NaCl, 0.1 mM EDTA, 0.1 mM EGTA, 0.1% NP-40, 1 mM DTT and a protease inhibitor cocktail. Following 20 sec of sonication on ice, the nuclear extract was obtained by centrifugation at 14,000 rpm for 10 min at 4°C. The protein concentrations of the cytoplasmic and nuclear preparations were determined as described.

### TBP/Q_61∼79_ aggregation assay

HEK-293T cells were plated in 12-well (1×10^5^/well) dishes, grown for 20 hr and transfected with TBP/Q_61_-GFP and *HMGB1* cDNA or pcDNA3 vector plasmids (1.5 µg each). After 48 hr, the cells were stained with Hoechst 33342 (0.1 µg/ml, Sigma), and the aggregation percentage was assessed using a high-content analysis (HCA) system (ImageXpressMICRO, Molecular Devices) with excitation/emission wavelengths at 482/536 (GFP).

### *HSPA5* promoter construct and dual luciferase assay

The *HSPA5*-luciferase reporter was constructed as previously described [29]. The 293-derived TBP/Q_36_ and TBP/Q_61_ cells [21] were plated in 24-well dishes (5×10^4^/well) and induced with doxycycline to express TBP. After 48 hr, the cells were co-transfected with the *HSPA5* luciferase reporter and *HMGB1* cDNA or pcDNA3 vector (2 µg each). The cells were grown for 48 hr. Cell lysates were prepared, and the luciferase activities of the lysates were measured as described [29]. Three independent transfection experiments were performed.

### ROS measurement

Intracellular superoxide and peroxide were measured using dihydroethidium (DHE) and 2′,7′-dichlorofluorescin diacetate (DCFH-DA) (Molecular Probes), respectively. In cells, DHE is oxidized by superoxides to the fluorescent substance ethidium, which then binds to DNA and further amplifies its fluorescence. In the cell, DCFH-DA is cleaved by esterases, yielding polarized nonfluorescent dichlorofluorescein (DCFH). Through peroxides, DCFH is oxidized to the fluorescent product dichlorofluorescein (DCF). The 293-derived TBP/Q_36∼79_ cells were grown for two days with doxycycline. DHE or DCFH-DA was diluted in fresh medium at a final concentration of 5 µM and incubated with the cells for 30 min at 37°C. The cells (10^5^) were washed once with PBS. A spectrofluorometer (SpectraMax Gemini XPS, Molecular Devices) with excitation/emission filters of 518/605 nm and 485/535 nm was used to detect ethidium and DCF, respectively. The concentration of DHE taken up by the cells was measured at excitation fluorescence 355 nm/emission 420 nm.

### Neuronal phenotype examination

A SH-SY5Y host cell line generated from independent integration of the pcDNA6/TR (a plasmid that expresses the Tet repressor and blasticidin-resistance genes) and pFRT/neomycin (a plasmid that contains the Flp Recombination Target (FRT) site and a neomycin-resistance gene (no ATG)) plasmids was used to establish the tetracycline-inducible TBP/Q_36∼79_ cell lines. The cells were plated in 6-well dishes (5×10^4^/well), and *trans* retinoic acid (10 µM, Sigma) was added at the time of seeding. On day 2, doxycycline (5 µg/ml) was added, and the cells were maintained in medium containing 10 µM *trans* retinoic acid and doxycycline for 10 days. The cells were subsequently stained with phalloidin (13.2 nM, Invitrogen). For HMGB1 knockdown, siRNA was electroporated into the SH-SY5Y cells (4D-Nucleofector System, Lonza). The morphologic differentiation, which included total outgrowth, processes and branches, was assessed using Metamorph microscopy automation and image analysis software (Molecular Devices).

### Statistical analysis

For each set of values, data were expressed as the mean ± standard deviation (SD) of three independent experiments. Differences between groups were analyzed by Student’s *t*-test or ANOVA (one-way and two-way) with *post-hoc* LSD test where appropriate. Values of *P*<0.05 were considered significant.
